# Assessment of three criteria to establish borrelial infection in suspected lyme neuroborreliosis

**DOI:** 10.1007/s15010-024-02338-2

**Published:** 2024-07-09

**Authors:** Katarina Ogrinc, Petra Bogovič, Vera Maraspin, Stanka Lotrič Furlan, Tereza Rojko, Eva Ružić-Sabljić, Andrej Kastrin, Klemen Strle, Gary P. Wormser, Franc Strle

**Affiliations:** 1 Department of Infectious Diseases, University Medical Center Ljubljana, Ljubljana, Slovenia; 2 Institute of Microbiology and Immunology, Faculty of Medicine, University of Ljubljana, Ljubljana, Slovenia; 3 Institute for Biostatistics and Medical Informatics, Faculty of Medicine, University of Ljubljana, Ljubljana, Slovenia; 4 Tufts University School of Medicine, Boston, Mass USA; 5 New York Medical College, Valhalla, NY USA

**Keywords:** Lyme neuroborreliosis, Intrathecal borrelial antibody synthesis, Erythema migrans, *Borrelia burgdorferi*, Culture of *Borrelia* from cerebrospinal fluid

## Abstract

**Purpose:**

Diagnosis of (European) Lyme neuroborreliosis has been based on clinical presentation, cerebrospinal fluid (CSF) pleocytosis and demonstration of intrathecal borrelial antibody synthesis (ITBAS) to document *Borrelia burgdorferi* s. l. infection. It is not known if other criteria to document *Borrelia* infection may contribute to the diagnosis.

**Methods:**

We compared the sensitivity of three individual criteria (ITBAS, CSF *Borrelia* culture, and the presence of erythema migrans [EM]) to confirm the diagnosis of early Lyme neuroborreliosis in 280 patients ≥ 15 years of age evaluated at a Lyme borreliosis outpatient clinic in Slovenia. The patients had either radicular pain of new onset or involvement of a cranial nerve but without radicular pain, each in conjunction with CSF pleocytosis. Evaluation was of patients who had each of the three confirmatory criteria assessed, and for whom at least one criterion was positive.

**Results:**

Analysis of 280 patients, 120 women and 160 men, median age 57 (range 15–84) years, revealed that ITBAS was the most frequently observed positive criterion (85.4%), followed by EM (52.9%), and by a positive CSF *Borrelia* culture (9.6%). Of the 280 patients, 154 (55%) met only one criterion (43.2% ITBAS only, 10.7% EM only, and 1.1% positive CSF culture only), whereas 42.1% met two criteria. Only 2.9% of patients were positive by all three criteria.

**Conclusion:**

Although ITBAS was the most frequent criterion for confirmation for *Borrelia* infection, the presence of EM alone confirmed an additional 10.7% of patients and a positive CSF *Borrelia* culture alone added another 1.1%.

## Introduction

Lyme neuroborreliosis (LNB) is the second most common clinical manifestation of disseminated Lyme borreliosis (LB) in Europe (after multiple erythema migrans [EM]) [1, 2]. The two most typical clinical presentations of early European LNB in adults are borrelial meningoradiculoneuritis (Bannwarth’s syndrome), which is clinically characterized by severe radicular pain; and borrelial cranial neuritis, most often manifesting as a peripheral facial nerve palsy [1, 2]. For the last 30 years at the LB Outpatient Clinic in Ljubljana, Slovenia, patients with clinically suspected LNB were offered a cerebrospinal fluid (CSF) examination. In addition, three criteria were used as evidence for *B. burgdorferi* s. l. infection: detection of intrathecal borrelial antibody synthesis (ITBAS), isolation of *B. burgdorferi* s. l. from CSF, and having an EM skin lesion currently or recently.

The objective of the present study was to compare the sensitivity of these three individual criteria for confirmation of early LNB in patients who had a clinical presentation consistent with LNB in conjunction with CSF pleocytosis.

## Patients and methods

This retrospective study was approved by the Medical Ethics Committee of the Ministry of Health of the Republic of Slovenia (0120–552/2023/3) and did not require written informed consent.

### Patients

The study used clinical data collected from 2005 to 2022 at the LB Outpatient Clinic of the Department of Infectious Diseases, UMC Ljubljana. All patients ≥ 15 years of age, who had: (i) radicular pain of new onset and CSF pleocytosis, or (ii) peripheral facial nerve palsy or involvement of other cranial nerves but without radicular pain, and who had CSF pleocytosis, and were assessed by all three confirmatory criteria for demonstration of *Borrelia* infection (ITBAS, isolation of *Borrelia* from CSF, and EM) and for whom at least one criterion was positive, qualified for inclusion. All patients when evaluated were asked about the presence of skin lesions and in addition, were carefully examined for an EM skin lesion.

### Laboratory evaluation

Routine blood and CSF laboratory testing was performed. In addition, immunoglobulin classes G and M (IgG and IgM, respectively) and albumin levels were determined in both serum and CSF. Up to 2011, antibodies to *B. burgdorferi* s. l. in serum and CSF were determined by an indirect immunofluorescence assay, using a local isolate of *B. afzelii.* Reactivity at serum dilutions of 1:256 or higher was interpreted as positive, based on the results of a control group from the same geographic region [3]. Since 2011, however, an indirect chemiluminescence immunoassay (LIAISON^®^, Diasorin, Italy), that incorporated the recombinant antigens OspC and VlsE, was used for detection of IgM antibody, whereas the VlsE antigen alone was used for detection of IgG antibody. Results were interpreted according to the manufacturer’s instructions. ITBAS was determined as described by Reiber and Peter [4].

Serum IgM and IgG antibodies to tick-borne encephalitis virus (TBEV) were assessed, as described previously [5]; patients with IgM antibodies to TBEV were excluded from this study.

Cultivation of *B. burgdorferi* s. l. from CSF was performed as described previously [6].

### Definitions

EM was defined as an expanding erythematous skin lesion, with or without central clearing, which develops days to weeks after a tick bite or exposure to ticks in a Lyme borreliosis endemic region and had a diameter of ≥ 5 cm [7]. In accordance with the European Federation of Neurological Societies (EFNS) guidelines for LNB [8], the diagnosis of possible early LNB was based on the following two criteria: Criterion #1) the presence of neurologic symptoms suggestive of LNB (with no other obvious explanation) with a duration of less than six months; Criterion #2) the presence of CSF pleocytosis (> 5 × 10^6^ leukocytes/L). For a diagnosis of confirmed early LNB the EFNS guideline requires detection of ITBAS. For patients in this study, however, the diagnosis of confirmed early LNB was based on either a positive ITBAS (IgM and/or IgG) or having one additional criterion: cultivation of *B. burgdorferi* s. l. from CSF, or the presence of a concomitant EM skin lesion (or a reliable history of EM within the prior three months).

### Statistical approaches

Continuous variables were summarized using median values and ranges or interquartile ranges (IQRs), and discrete variables using frequencies and percentages (with 95% confidence intervals, CIs). The Fisher exact test was used to compare differences in categorical variables. Differences in the duration (in days) of neurologic symptoms were compared using the Wilcoxon rank-sum test. A p-value < 0.05 was considered statistically significant. Multiple logistic regression models were applied to estimate odds ratios (with 95% CIs) for each of the four outcomes: (a) Patients with meningoradiculoneuritis vs. patients with cranial neuritis without radicular pain; (b) Patients with meningoradiculoneuritis with cranial neuritis vs. patients with meningoradiculoneuritis without cranial neuritis; (c) Patients with meningoradiculoneuritis with cranial neuritis vs. patients with cranial neuritis without radicular pain; and (d) Patients with meningoradiculoneuritis without cranial neuritis vs. patients with cranial neuritis without radicular pain. The Benjamini and Hochberg procedure for correcting false positives due to multiple comparisons was used to adjust the p values for the regressions performed. All statistical analyses were performed using R software (v. 4.4.0) [9].

## Results

**Fig. 1:**
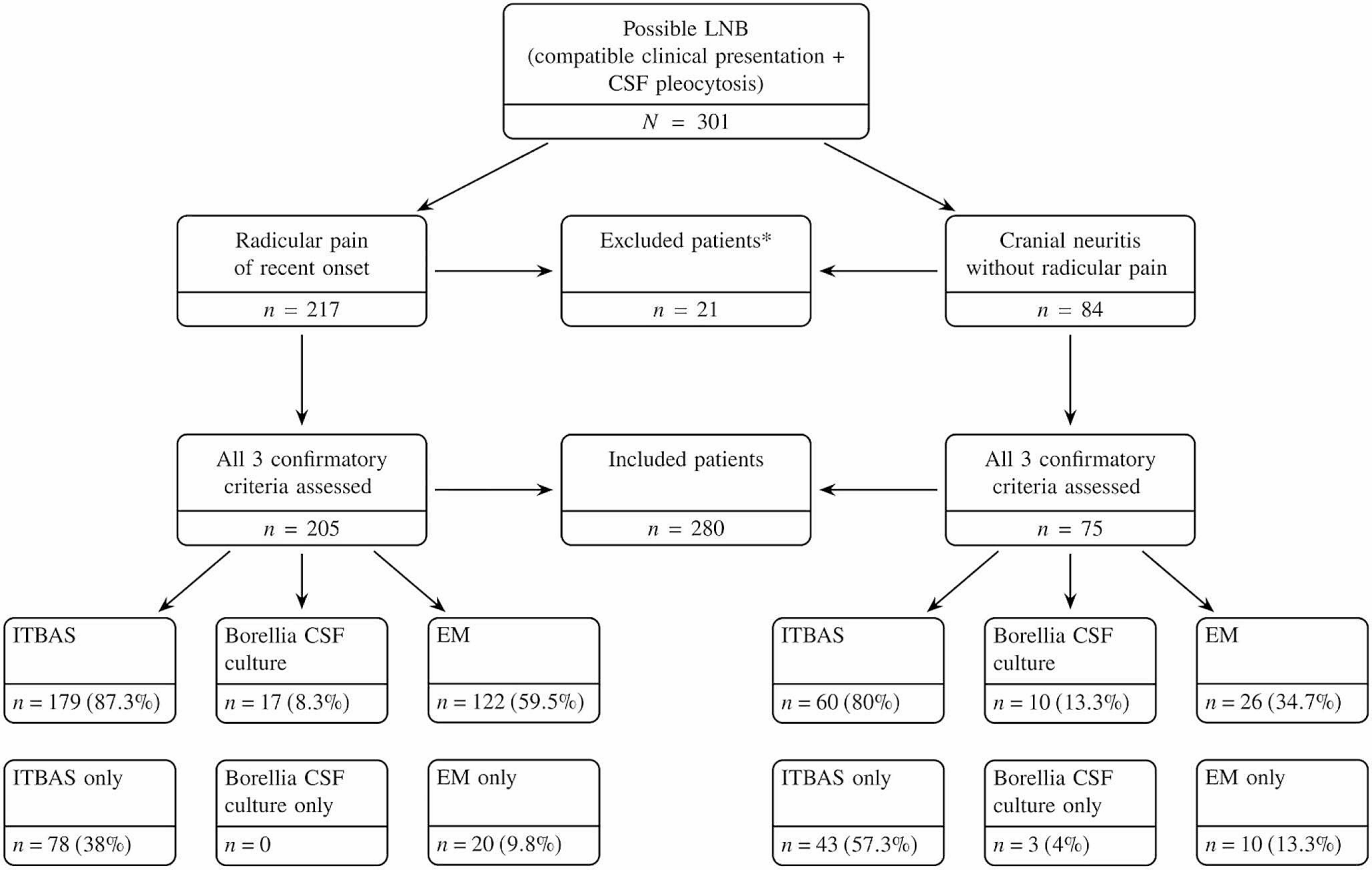
Title: Patients evaluated for the assessment of sensitivity of three criteria for demonstration of *Borrelia* infection: intrathecal borrelial antibody synthesis (ITBAS), isolation of *Borrelia* from cerebrospinal fluid (CSF), and erythema migrans (EM). Abbreviations: LNB, Lyme neuroborreliosis; CSF, cerebrospinal fluid; ITBAS, intrathecal borrelial antibody synthesis; EM, erythema migrans. *Patients were excluded, because all three confirmatory criteria were not assessed (CSF *Borrelia* culture was not performed)

Three hundred one patients ≥ 15 years of age who had radicular pain and/or cranial neuritis without another obvious explanation and had CSF pleocytosis were assessed for LNB at our LB Outpatient Clinic in Slovenia in the period 2005 − 2022. Twenty-one patients out of the 301 were excluded due to a missing CSF *Borrelia* culture, while 280 patients, 120 (42.9%) women and 160 (57.1%) men, median age 57 (range 15–84) years, who had been assessed by all three confirmatory criteria for LNB and had at least one positive criterion qualified for inclusion in this study. Two hundred five presented with radicular pain of recent onset and 75 with cranial neuritis without radicular pain (Fig. 1; Table 1).

**Table 1:** Comparison of diagnostic test results in 280 cases of Lyme neuroborreliosis with or without radicular pain

Tests	Borrelial Meningoradiculoneuritis			Borrelial CN without radicular pain *n* = 75 Column #4	*P*-value 1 1 vs. 4	*P*-value 2 2 vs. 3	*P*-value 3 2 vs. 4	*P*-value 4 3 vs. 4
	All *n* = 205 Column #1	With CN *n* = 76 Column #2	Without CN *n* = 129 Column #3					
Male sex	111 (54.1)	45 (79.2)	66 (51.2)	49 (65.3)	0.124	0.331	0.543	0.069
Age*	59 (21–84)	58 (21–82)	60 (21–84)	46 (15–79)	**< 0.001**	0.201	**0.002**	**< 0.001**
All 3 tests +	6 (2.9, 1.1–6.3)	1 (1.3, 0–7.1)	5 (3.9, 1.3–8.8)	2 (2.7, 0.3–9.3)	> 0.999	0.416	0.620	> 0.999
Only ITBAS +	78 (38.0, 31.4–45.1)	48 (63.2, 51.3–73.9)	30 (23.3, 16.3–31.5)	43 (57.3, 45.4–68.7)	**0.004**	**< 0.001**	0.508	**< 0.001**
Only EM	20 (9.8, 6.1–14.7)	2 (2.6, 0.3–9.2)	18 (14.0, 8.5–21.2)	10 (13.3, 6.6–23.2)	0.389	**0.007**	**0.017**	> 0.999
Only culture +	0 (0, 0–1.8)	0 (0, 0–4.7)	0 (0, 0–2.8)	3 (4.0, 0.8–11.3)	**0.019**	NA	0.120	**0.048**
2 of 3 tests +	101 (49.3, 42.2–56.3)	25 (32.9, 22.5–44.6)	76 (58.9, 49.9–67.5)	17 (22.7, 13.8–33.8)	**< 0.001**	**< 0.001**	0.204	**< 0.001**
ITBAS + plus EM	90 (43.9, 37.0–51.0)	22 (28.9, 19.1–40.5)	68 (52.7, 43.7–61.6)	12 (16.0, 8.6–26.3)	**< 0.001**	**0.001**	0.079	**< 0.001**
Culture + plus EM	6 (2.9, 1.1–6.3)	0 (0, 0–4.7)	6 (4.7, 1.7–9.9)	2 (2.7, 0.3–9.3)	> 0.999	0.087	0.245	0.713
ITBAS + plus Culture +	5 (2.4, 0.8–5.6)	3 (4.0, 0.8–11.1)	2 (1.6, 0.2–5.5)	3 (4.0, 0.8–11.3)	0.445	0.362	> 0.999	0.359
ITBAS +	179 (87.3, 82.0–91.6)	74 (97.4, 90.8–99.7)	105 (81.4, 73.6–87.7)	60 (80.0, 69.2–88.4)	0.131	**0.001**	**0.001**	0.854
Culture +	17 (8.3, 4.9–13.0)	4 (5.3, 1.5–12.9)	13 (10.1, 5.5–16.6)	10 (13.3, 6.6–23.2)	**0.038**	0.298	0.100	0.498
EM	122 (59.5, 52.5–66.3)	25 (32.9, 22.5–44.6)	97 (75.2, 66.8–82.4)	26 (34.7, 24.0–46.5)	**< 0.001**	**< 0.001**	0.864	**< 0.001**
Duration (days) of neurologic symptoms^a^	22 (14–42)	26 (16–41)	21 (10–45)	10 (4–21)	**0.004**	0.202	**< 0.001**	**0.002**
Borrelial antibodies in serum	190 (92.7, 88.2–95.9)	71 (93.4, 85.3–97.8)	119 (92.2, 86.2–96.2)	61 (81.3, 70.7–89.4)	**0.011**	0.973	**0.046**	**0.035**
Borrelial antibodies in serum in the absence of ITBAS	18/26 (69.2; 48.2–85.7)	1/2 (50.0; 1.3–98.7)	17/24 (70.8; 48.9–87.4)	9/15 (60.0; 32.3–83.7)	0.796	0.529	0.669	0.727
Previous LB	39 (19.0, 13.9–25.1)	11 (14.5, 7.5–24.4)	28 (21.7, 14.9–29.8)	7 (9.3, 0–0.2)	0.079	0.276	0.469	**0.039**
Previous antibiotic treatment^b^	41^c^ (20.0, 14.8–26.1)	9^d^ (11.8, 5.6–21.3)	32^e^ (24.8, 17.6–33.2)	15^f^ (20.0, 11.7–30.8)	0.866	**0.039**	0.251	0.539
CSF culture + in antibiotic treated patients^g^	3/41 (7.3, 1.5–19.9)	1/9 (11.1, 0.3–48.3)	2/32 (6.3, 0.8–20.8)	1/15 (6.7, 0.2–32.0)	0.711	0.535	0.620	0.694
CSF culture + in antibiotic untreated patients^h^	14/164 (8.5, 4.8‒13.9)	3/67 (4.5, 9.3‒12.5)	11/97 (11.3, 5.8‒19.4)	9/60 (15.0, 7.1‒26.6)	0.245	0.207	0.085	0.673
ITBAS + in antibiotic treated patients^i^	34/41 (82.9, 67.9‒92.9)	8/9 (88.9, 51.8‒99.7)	26/32 (81.3, 63.6‒92.8)	10/15 (66.7, 38.4‒88.2)	0.171	0.512	0.238	0.229
ITBAS + in antibiotic untreated patients^j^	145/164 (88.4, 82.5‒92.9)	66/67 (98.5, 92.0‒100)	79/97 (81.4, 72.3‒88.6)	50/60 (83.3, 71.5‒91.7)	0.436	**0.002**	**0.007**	0.931

Data shown as frequencies (%, 95% CI for population proportion). Boldface is used for p-values < 0.05

Abbreviations: CN, cranial neuritis; +, positive; ITBAS, intrathecal *Borrelia* antibody synthesis index; EM, erythema migrans; NA, not applicable; LB, Lyme borreliosis; CSF, cerebrospinal fluid

*Reported as median (range)

^a^At testing

^b^Antibiotics to which *Borrelia* are susceptible in vitro or unknown antibiotic prescribed for treatment of EM.

^c^Azithromycin in 38 patients (3 g in 33, < 3 g or unknown dose in 5), doxycycline for 7 days, amoxicillin for 2 days, and unknown antibiotic, each in 1 patient

^d^Azithromycin in 8 patients (3 g in 7, unknown dose in 1), unknown antibiotic in 1 patient

^e^Azithromycin in 30 patients (3 g in 26, < 3 g or unknown dose in 4), doxycycline for 7 days, and amoxicillin for 2 days, each in 1 patient

^f^Azithromycin in 8 patients (3 g in 5, < 3 g or unknown dose in 3), amoxicillin for < 14 days in 4 patients, clarithromycin for 7 days, doxycycline for 2 days + azithromycin 1.5 g, and unknown antibiotic, each in 1 patient

^g,h^Comparison of CSF culture positivity between antibiotic treated (g) and untreated patients (h) revealed no significant differences in any of the group (*P* = 0.403–1.0)

^i,j^Comparison of ITBAS positivity between antibiotic treated (i) and untreated patients (j) revealed no significant differences in any of the group (*P* = 0.164–1.0)

Comparisons: *P* value 1: All Borrelial Meningoradiculoneuritis cases vs. Borrelial CN cases without radicular pain

P-value 2: Borrelial Meningoradiculoneuritis cases with CN vs. Borrelial Meningoradiculoneuritis cases without CN

P-value 3: Borrelial Meningoradiculoneuritis cases with CN vs. Borrelial CN cases without radicular pain

P-value 4: Borrelial Meningoradiculoneuritis cases without CN vs. Borrelial CN cases without radicular pain

The Fisher exact test was used to compare differences in categorical variables. Differences in the duration (in days) of neurologic symptoms were compared using the Wilcoxon rank-sum test

ITBAS was demonstrated in 239 (85.4%), EM in 148 (52.9%), and *B. burgdorferi* s. l. was cultured from CSF in 27 (9.6%) cases. ITBAS was the sole positive criterion in 121 patients (43.2%), EM skin lesion in 30 (10.7%) patients, and a positive CSF culture in three (1.1%) patients (Fig. 1; Table 1).

Of 27 *Borrelia* CSF culture positive patients, 16 were ITBAS positive (8 ITBAS + with a + culture, 8 ITBAS + with a + culture and with EM), while 11 were ITBAS negative. *Borrelia* was isolated from CSF significantly more often in ITBAS negative patients than in ITBAS positive patients: 11/41 (26.8%, 95% CI 14.2–42.9%) versus 16/239 (6.7%, 95% CI 3.9 − 10.6%; *p* = 0.002).

56/280 patients (20%) had received at least one dose of an antibiotic during the course of their illness prior to diagnostic testing for LNB (Table 1). In comparison to previously untreated patients, those who had received antibiotic therapy had a lower *Borrelia* CSF culture positivity rate (4/56, 7.1% vs. 23/224, 10.3%; *p* = 0.617) and lower proportion of a + ITBAS (44/56, 78.6% vs. 195/224, 87.1%; *p* = 0.137); however, the differences were not statistically significant. All four previously treated patients with isolation of *Borrelia* from CSF had received azithromycin within four weeks before the culture and other diagnostic testing for LNB.

### Borrelial meningoradiculoneuritis

Two hundred five patients diagnosed with borrelial meningoradiculoneuritis had been assessed using the three confirmatory diagnostic criteria for *B. burgdorferi* s. l. infection. Of these patients, 94 (45.9%) were female and 111 (54.1%) were male, with an overall median age of 59 (range 21–84) years. In addition to meningoradiculoneuritis, 76 (37.0%) of these 205 patients also had a cranial neuropathy. ITBAS was demonstrated in 179 (87.3%), EM in 122 (59.5%), and *B. burgdorferi* s. l. was cultured from CSF in 17 (8.3%). Only six cases (2.9%) were positive, however, by all three criteria. ITBAS was the sole positive criterion in 78 patients (38.0%), an EM skin lesion was the sole positive criterion in 20 (9.8%) patients, whereas none of the patients had CSF culture positivity as the sole criterion, *p* < 0.001.

One hundred one (49.3%) of these cases met exactly two of the three confirmatory criteria, including 90 (43.9%) who had both ITBAS and EM, six (2.9%) who were culture positive and had EM, and five (2.4%) who had ITBAS and were culture positive, *p* < 0.001 (Table 1).

### Cranial neuritis without radicular pain

During the same 18-year time period, 75 patients without radicular pain were diagnosed with early LNB manifested by cranial neuropathy along with CSF pleocytosis: 26 (34.7%) women, 49 (65.3%) men; median age 46 (15–79) years. All but six had peripheral facial nerve palsy. These 75 patients were assessed for all three of the confirmatory diagnostic criteria to document *B. burgdorferi* s. l. infection: ITBAS was demonstrated in 60 (80.0%), EM in 26 (34.7%), while for 10 patients (13.3%) *B. burgdorferi* s. l. was isolated from CSF. ITBAS was the sole positive criterion for 43 (57.3%). Having an EM skin lesion was the sole positive criterion for 10 (13.3%), whereas CSF culture positivity was the only positive criterion for three of the cases (4.0%), *p* < 0.001. Seventeen (22.7%) of the cases met exactly two of the three confirmatory criteria, including 12 (16.0%) who had both ITBAS and EM, two (2.7%) who were culture positive and had EM, and three (4.0%) who were ITBAS positive and culture positive, *p* < 0.001. Only two (2.7%) patients were positive by all three confirmatory criteria (Table 1).

### Comparisons

Comparison of the positivity rate for each of the three confirmatory diagnostic criteria (ITBAS, *Borrelia* CSF culture, the presence of an EM skin lesion) in the 280 cases of LNB, manifesting as either radiculopathy (with or without cranial neuritis) or cranial neuropathy without radicular pain, for whom all three tests were performed, is shown in Table 1.

Patients with borrelial meningoradiculoneuritis (that included 76 patients who also had a cranial neuropathy) and patients with cranial neuritis without radicular pain significantly differed in the proportions testing positive for the three individual confirmatory criteria. Patients with borrelial meningoradiculoneuritis more often had EM (122/205, 59.5% vs. 26/75, 34.7%, *p* < 0.001) but less often had *B. burgdorferi* s. l. isolated from CSF (17/205, 8.3% vs. 10/75, 13.3%; *p* = 0.038). Patients with borrelial meningoradiculoneuritis also more frequently had two of the three confirmatory criteria (101/205, 49.3% vs. 17/75, 22.7%; *p* < 0.001), including the combination of ITBAS plus EM (90/205, 43.9% vs. 12/75, 16.0%; *p* < 0.001). Furthermore, when only one criterion was fulfilled, patients with meningoradiculoneuritis (*n* = 98) less often had a positive CSF culture, compared to patients with cranial neuritis without radicular pain (*n* = 56): 0/98 vs. 3/56, 5.4%; *p* = 0.021.

Of the 205 patients with borrelial meningoradiculoneuritis, 76 (37.1%) also had cranial neuritis (all but one of these patients had peripheral facial nerve palsy). Comparison of the subgroup of 76 patients with borrelial meningoradiculoneuritis who had cranial neuritis, with the subgroup of 129 patients without cranial neuritis revealed several statistically significant differences. In patients with cranial neuritis, ITBAS was more frequently present (74/76, 97.4% vs. 105/129, 81.4%; *p* = 0.001), but this subgroup less often had EM (25/76, 32.9% vs. 97/129, 75.2%; *p* < 0.001). Additionally, the subgroup with borrelial meningoradiculoneuritis who had cranial neuritis more often had a positive ITBAS as the sole confirmatory criterion (48/76, 63.2% vs. 30/129, 23.3%; *p* < 0.001) and less often had EM as the sole confirmatory criterion (2/76, 2.6% vs. 18/129, 14.0%; *p* = 0.007), and less often had exactly two positive criteria (25/76, 32.9% vs. 76/129, 58.9%; *p* < 0.001), including the combination of ITBAS plus EM (22/76, 28.9% vs. 68/129, 52.7%; *p* = 0.001).

As shown in Table 1, patients with borrelial meningoradiculoneuritis who had cranial neuritis were more similar to patients with cranial neuritis without radicular pain than to patients with meningoradiculoneuritis but without cranial neuritis, in regard to the frequency of these three confirmatory criteria. A comparison of patients with borrelial cranial neuritis without radicular pain with the subgroup of patients with meningoradiculoneuritis who had no cranial nerve involvement revealed that out of the 11 tested parameters five were statistically significantly different (the presence of EM: 26/75, 34.7% vs. 97/129, 75.2%, *p* < 0.001; ITBAS as the sole confirmatory diagnostic criterion: 43/75, 57.3% vs. 30/129, 23.3%, *p* < 0.001; a positive CSF culture as the sole confirmatory diagnostic criterion: 3/75, 4.0% vs. 0/129, 0%, *p* = 0.048; positivity for two out of three confirmatory diagnostic criteria: 17/75, 22.7% vs. 76/129, 58.9%, *p* < 0.001, including the combination of ITBAS plus EM: 12/75, 16.0% vs. 68/129, 52.7%, *p* < 0.001). In contrast, only two significant differences were observed when comparing patients with borrelial cranial neuritis without radicular pain with the subgroup of patients who had meningoradiculoneuritis associated with cranial nerve involvement (the presence of ITBAS: 60/75, 80% vs. 74/76, 97.4%, *p* = 0.001; and EM as the only diagnostic criterion: 10/75, 13.3% vs. 2/76, 2.6%, *p* = 0.017).

As shown in Table 2, several of these differences were also found using multivariate regression analyses. Multiple logistic regression models revealed that in comparison to patients with cranial neuritis without radicular pain, patients with borrelial meningoradiculoneuritis were significantly older, had longer duration of neurologic symptoms at diagnosis, and had 5.2-times higher odds for the presence of EM, while associations for several other tested parameters were not statistically significant (Table 2).

**Table 2:** Multiple logistic regression of tests/characteristics of patients with borrelial meningoradiculoneuritis and patients with cranial neuritis without radicular pain

Characteristic/Test	OR (95% CI)	*p*_raw_	*p*_adj_
**Sex (male)**			
Borrelial MR (All) vs. Borrelial CN (without RP)	0.90 (0.46, 1.73)	0.748	0.831
Borrelial MR (with CN) vs. Borrelial MR (without CN)	1.00 (0.50, 1.98)	0.995	0.995
Borrelial MR (with CN) vs. Borrelial CN (without RP)	0.95 (0.45, 2.02)	0.887	0.956
Borrelial MR (without CN) vs. Borrelial CN (without RP)	1.16 (0.52, 2.62)	0.717	0.797
**Age**			
Borrelial MR (All) vs. Borrelial CN (without RP)	1.03 (1.01, 1.06)	**0.002**	**0.006**
Borrelial MR (with CN) vs. Borrelial MR (without CN)	0.99 (0.97, 1.02)	0.494	0.988
Borrelial MR (with CN) vs. Borrelial CN (without RP)	1.03 (1.01, 1.06)	**0.005**	**0.023**
Borrelial MR (without CN) vs. Borrelial CN (without RP)	1.04 (1.01, 1.07)	**0.004**	**0.011**
**ITBAS (+)**			
Borrelial MR (All) vs. Borrelial CN (without RP)	3.11 (1.08, 8.88)	**0.035**	0.070
Borrelial MR (with CN) vs. Borrelial MR (without CN)	3.95 (0.89, 37.55)	0.075	0.250
Borrelial MR (with CN) vs. Borrelial CN (without RP)	11.06 (2.01, 116.19)	**0.004**	**0.023**
Borrelial MR (without CN) vs. Borrelial CN (without RP)	2.65 (0.90, 7.82)	0.077	0.129
**Culture (+)**			
Borrelial MR (All) vs. Borrelial CN (without RP)	1.05 (0.36, 3.51)	0.927	0.927
Borrelial MR (with CN) vs. Borrelial MR (without CN)	0.72 (0.19, 2.39)	0.596	0.994
Borrelial MR (with CN) vs. Borrelial CN (without RP)	0.79 (0.19, 3.29)	0.736	0.920
Borrelial MR (without CN) vs. Borrelial CN (without RP)	1.12 (0.34, 4.26)	0.854	0.854
**EM**			
Borrelial MR (All) vs. Borrelial CN (without RP)	5.17 (2.33, 12.46)	**< 0.001**	**< 0.001**
Borrelial MR (with CN) vs. Borrelial MR (without CN)	0.18 (0.08, 0.37)	**< 0.001**	**< 0.001**
Borrelial MR (with CN) vs. Borrelial CN (without RP)	2.07 (0.83, 5.61)	0.121	0.242
Borrelial MR (without CN) vs. Borrelial CN (without RP)	12.22 (4.85, 33.90)	**< 0.001**	**< 0.001**
**Duration (days) of neurologic symptoms**			
Borrelial MR (All) vs. Borrelial CN (without RP)	1.03 (1.01, 1.05)	**0.001**	**0.003**
Borrelial MR (with CN) vs. Borrelial MR (without CN)	0.98 (0.97, 1.00)	**0.031**	0.157
Borrelial MR (with CN) vs. Borrelial CN (without RP)	1.01 (1.00, 1.04)	0.106	0.242
Borrelial MR (without CN) vs. Borrelial CN (without RP)	1.02 (1.01, 1.04)	**0.001**	**0.006**
**Borrelial antibodies in serum**			
Borrelial MR (All) vs. Borrelial CN (without RP)	1.53 (0.10, 23.03)	0.745	0.831
Borrelial MR (with CN) vs. Borrelial MR (without CN)	0.92 (0.01, 20.15)	0.962	0.995
Borrelial MR (with CN) vs. Borrelial CN (without RP)	1.07 (0.07, 16.34)	0.956	0.956
Borrelial MR (without CN) vs. Borrelial CN (without RP)	2.48 (0.12, 380.13)	0.572	0.715
**Previous LB**			
Borrelial MR (All) vs. Borrelial CN (without RP)	1.82 (0.73, 5.17)	0.210	0.350
Borrelial MR (with CN) vs. Borrelial MR (without CN)	0.53 (0.22, 1.23)	0.142	0.354
Borrelial MR (with CN) vs. Borrelial CN (without RP)	0.80 (0.25, 2.64)	0.705	0.920
Borrelial MR (without CN) vs. Borrelial CN (without RP)	2.91 (1.04, 9.23)	0.042	0.084
**Previous antibiotic treatment**			
Borrelial MR (All) vs. Borrelial CN (without RP)	0.57 (0.22, 1.48)	0.245	0.350
Borrelial MR (with CN) vs. Borrelial MR (without CN)	0.98 (0.36, 2.49)	0.960	0.995
Borrelial MR (with CN) vs. Borrelial CN (without RP)	0.51 (0.14, 1.73)	0.282	0.470
Borrelial MR (without CN) vs. Borrelial CN (without RP)	0.60 (0.22, 1.66)	0.318	0.455

Boldface is used for p-values < 0.05

Abbreviations: MR, meningoradiculoneuritis; CN, cranial neuritis; RP, radicular pain; ITBAS, intrathecal *Borrelia *antibody synthesis index; +, positive; EM, erythema migrans; LB, Lyme borreliosis; OR, odds ratio; CI, confidence interval; p_raw_, p value; p_adj_, p value adjusted for multiple comparisons

The only striking finding in comparison of the subgroups with and without cranial neuritis within the borrelial meningoradiculoneuritis group was that those with cranial neuritis had a 5.6 times lower odds for having EM than those without cranial neuritis (Table 2).

Comparison of patients who had cranial neuritis without radicular pain with the subgroup of patients with meningoradiculoneuritis, who also had cranial neuritis, revealed that the latter group was significantly older and had 11.1-times higher odds for having ITBAS. A comparison of patients who had cranial neuritis without radicular pain with the subgroup of patients with meningoradiculoneuritis without cranial neuritis demonstrated a 12.2-times higher odds for having EM, in addition to older age, for the meningoradiculoneuritis group (Table 2).

## Discussion

The objective of the present study was to determine the contribution of each of the three individual confirmatory criteria (ITBAS, isolation of *B. burgdorferi* s. l. from CSF, and the presence of EM) to LNB diagnosis, and to compare these criteria in patients with different clinical presentations of early LNB. The present study consisted of 280 patients with CSF pleocytosis that included two clinical subgroups: 205 with borrelial meningoradiculoneuritis (with or without cranial nerve involvement) and 75 with cranial nerve involvement without radicular pain. We chose these two groups to evaluate because they represent the most common clinical manifestations of LNB in Europe [1, 2].

Multiple regression analyses showed that patients with borrelial meningoradiculoneuritis were older and had a longer duration of neurologic symptoms than those with cranial neuritis without radicular pain; in both groups males predominated, however, with no significant differences in sex between the two groups.

For the entire group of 280 patients with LNB, the most frequent confirmatory criterion was ITBAS (239/280, 85.4%), followed by EM (148/280, 52.9%) and by isolation of *B. burgdorferi* s. l. from CSF (27/280, 9.6%). All three diagnostic criteria were fulfilled in only 8/280 (2.9%) patients, primarily due to the small number of cases with a positive CSF culture. Besides low sensitivity, culturing the organism from CSF has several additional drawbacks: the procedure is not usually available, it requires a considerable work-burden and is time consuming with a substantial delay in getting the test results. Furthermore, CSF culture is very rarely the only positive diagnostic criterion (3/280, 1.1%): as was found in 0/205 (0%, 95 CI 0.0–1.8) patients with meningoradiculoneuritis and in 3/75 (4.0%, 95% CI 0.8–11.3) patients with cranial neuritis without radicular pain. However, culture positivity is highly specific and genetic analysis of the recovered isolates of *B. burgdorferi* s. l. might provide insights into the pathogenesis of LNB [10]. It is of interest that *Borrelia* was isolated from CSF significantly more often in ITBAS negative patients than in ITBAS positive patients (11/41, 26.8% versus 16/239, 6.7%; *p* = 0.002), suggesting an inhibitory role of borrelial antibodies in CSF.

Of the three confirmatory criteria evaluated in the present study, ITBAS was the most frequently observed positive test. ITBAS is the only criterion proposed by the EFNS guidelines [8] for diagnosing of borrelial infection in patients with suspected LNB. The main limiting factors in its use (and in interpretation of the test results) in clinical practice are the time delay in terms of duration of illness until intrathecal antibodies to *B. burgdorferi* s. l. develop, which may require several weeks, and the time delay in disappearance (it may take several months to several years for a positive ITBAS to become negative in recovered patients who had been appropriately treated with antibiotics) [1, 11].

Documentation of EM is a simple, non-expensive and reliable clinical indicator of a recent Lyme borrelia infection. Although it is not per se a specific marker of nervous system infection, it can significantly contribute to confirming the diagnosis of early LNB. Namely, a patient with a compatible neurologic presentation, who has CSF pleocytosis (a marker of active inflammation) and also has EM can be reliably interpreted to have LNB. EM should be carefully searched for in patients with suspected LNB with targeted questions about the presence of skin lesions and with a full body skin examination. In the current study, of the 122 patients who fulfilled criteria for borrelial meningoradiculoneuritis and had an associated EM skin lesion, for 11 (9.0%) the skin lesion was first discovered at the time of the visit for suspected LNB. A typical EM skin lesion is diagnostically of greater value than a positive serologic test for borrelial antibodies, which may be the result of either a recent or a remote borrelial infection, including a prior asymptomatic or self-resolved symptomatic infection. Asymptomatic infections are more common in Europe (approximately 60%) than in North America (10%) [12–17].

It is of interest that EM was present more often in patients with meningoradiculoneuritis than in those having cranial neuritis without radicular pain (OR = 5.2). This difference was even greater within the overall group of patients with meningoradiculoneuritis (i.e., the subgroup with cranial nerve involvement significantly less frequently had EM compared with those without cranial neuritis; OR = 0.18; *p* < 0.001), and comparing the subgroup of patients with borrelial meningoradiculoneuritis without cranial neuritis with those who had cranial neuritis without radicular pain (OR = 12.2, *p* < 0.001). These differences suggest differences in the pathogenesis of LNB in patients with, versus those without, cranial neuritis. Of interest, EM was the sole confirmatory criterion in 30 (10.7%) of the 280 patients with LNB. The presence of EM added 9.8% to confirming the diagnosis of borrelial meningoradiculoneuritis and 13.3% to confirming the diagnosis of borrelial cranial neuritis.

Previously reported information on the presence of all three criteria for borrelial infection in patients with suspected LNB is lacking, but there are reports on the frequency of the individual criteria. Case series including > 25 patients with clinically suspected LNB, with CSF pleocytosis, showed the presence of ITBAS in 56–96% [5, 18–25], EM in 17–60% [5, 24, 26–29], and successful isolation of *Borrelia* from CSF in 8–16% [5, 24, 28, 30, 31]. Although differences in inclusion criteria and methodology among the studies make comparisons difficult, nevertheless, the reported proportions are quite similar to those found in the present study, i.e., 85.4% for ITBAS, 52.9% for EM and 9.6% for the isolation of *Borrelia* from CSF.

The strengths of our study are the well-defined groups of patients with LNB and the large number of LNB cases, all of whom had CSF pleocytosis and for whom we used the same diagnostic approaches to confirm *B. burgdorferi* s. l. infection. However, there are several limitations. Our results apply to patients at least 15 years of age in Europe, but not necessarily to young children, for whom the course of LNB is somewhat different than in adults. In addition, these results may not pertain to North America, where borrelial meningoradiculoneuritis is rare [32], presumably because Lyme borreliosis in Europe is typically caused by different *Borrelia* species compared with North America.

In conclusion, assessment of 280 patients with a clinical presentation suggestive of LNB, all of whom had CSF pleocytosis, revealed that ITBAS was the most frequently observed positive criterion (85.4%), followed by EM (52.9%) and by isolation of Lyme borrelia from CSF (9.6%). For less than 3% of patients were all three criteria present, while 55.0% had only one criterion fulfilled (43.2% ITBAS only, 10.7% EM only, and 1.1% positive CSF culture only). The frequency of positivity for the three confirmatory criteria evaluated differed between distinct clinical presentations: patients who had meningoradiculoneuritis had EM significantly more often than those having cranial neuritis without radicular pain. This difference was also confirmed within the group of patients with meningoradiculoneuritis: the subgroup with cranial neuritis had EM less frequently than those without cranial nerve involvement. However, the presence of EM per se added only 9.8% to confirming the diagnosis of borrelial meningoradiculoneuritis and only 13.3% to confirming the diagnosis of borrelial cranial neuritis. The contribution of the isolation of Lyme borrelia from CSF was even lower than these values.

## Data Availability

All relevant data are presented in the manuscript. More detailed data can be provided by the authors upon reasonable request.
